# Cost-Effectiveness of Universal Prophylaxis in Pregnancy with Prior Group B Streptococci Colonization

**DOI:** 10.1155/2009/934698

**Published:** 2009-12-13

**Authors:** Mark A. Turrentine, Mildred M. Ramirez, Joan M. Mastrobattista

**Affiliations:** Department of Obstetrics, Gynecology and Reproductive Sciences, Kelsey Research Foundation, University of Texas Medical School, Houston, TX 77007, USA

## Abstract

*Objective*. To estimate the costs and outcomes of rescreening for group B streptococci (GBS) compared to universal treatment of term women with history of GBS colonization in a previous pregnancy. *Study Design*. A decision analysis model was used to compare costs and outcomes. Total cost included the costs of screening, intrapartum antibiotic prophylaxis (IAP), treatment for maternal anaphylaxis and death, evaluation of well infants whose mothers received IAP, and total costs for treatment of term neonatal early onset GBS sepsis. *Results*. When compared to screening and treating, universal treatment results in more women treated per GBS case prevented (155 versus 67) and prevents more cases of early onset GBS (1732 versus 1700) and neonatal deaths (52 versus 51) at a lower cost per case prevented ($8 805 versus $12 710). *Conclusion*. Universal treatment of term pregnancies with a history of previous GBS colonization is more cost-effective than the strategy of screening and treating based on positive culture results.

## 1. Introduction

Intrapartum infection with group B streptococci (GBS) may lead to untoward neonatal sequelae such as pneumonia, septicemia, and meningitis [1]. Several clinical trials have demonstrated that the use of intrapartum intravenous antibiotic prophylaxis is highly effective to prevent early onset neonatal GBS infections [1]. In August 2002, national prevention guidelines were released from the Centers for Disease Control and Prevention (CDC), the American Academy of Pediatrics (AAP), and the American College of Obstetricians and Gynecologists (ACOG) that recommend universal antenatal culture-based screening at 35 to 37 weeks of gestation to direct treatment for the prevention of early onset GBS disease [1, 2]. Since colonization can be variable, the above entities recommend that practitioners screen for GBS colonization in every pregnancy and that GBS colonization in a previous pregnancy is not an indication for intrapartum prophylaxis [1].

Recent longitudinal studies have demonstrated that women colonized with GBS during pregnancy are at an increased risk of colonization in a subsequent pregnancy [3, 4]. Reported rates of colonization range from 38% to 53% in subsequent pregnancies. Since many women positive for GBS during one pregnancy remain colonized in a subsequent pregnancy, it raises the question: Would it be cost-effective to universally administer intrapartum antibiotic prophylaxis to all women with a history of prior GBS colonization in a subsequent pregnancy? The aim of our study is to estimate whether it is cost-effective to base intrapartum GBS prophylaxis on rescreening versus universal treatment of all women with prior GBS colonization to prevent early onset GBS disease. 

## 2. Materials and Methods

**Figure 1:**
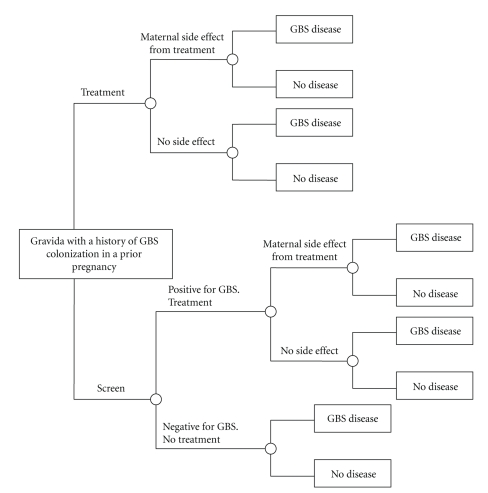
Decision-analysis model for evaluating the cost-effectiveness of screening-directed versus universal treatment of women with group B streptococci (GBS) colonization in a previous pregnancy on prevention of early onset neonatal GBS sepsis.

We constructed a decision-analysis model to evaluate the cost-effectiveness of screening-directed versus universal treatment for the prevention of early onset neonatal GBS sepsis in women colonized with GBS in a previous pregnancy. The pathways of events are shown in Figure 1. Since our objective is to compare screening-directed versus direct treatment, we limited our cost analysis to term gestations (37 weeks and above) and excluded women with indications for intrapartum prophylaxis (previous neonate with invasive GBS disease, GBS bacteriuria in the current pregnancy, and unknown GBS status in conjunction with any of the following amniotic membrane rupture for 18 hours or more and intrapartum temperature 38.0°C or more) [1]. Since current recommendations are for culture-based screening at 35 to 37 weeks of gestation, we did not perform a subgroup analysis for a rapid GBS test result at presentation in labor [1]. 

### 2.1. Strategies Analyzed

We compared two strategies described in the literature [1, 5]. With the screening strategy, all women are cultured for GBS colonization by vaginal-rectal swab at 35 to 37 weeks of gestation and those with a positive result receive intrapartum prophylaxis [1]. The universal treatment arm involved administering intrapartum prophylaxis to all previously colonized women when they presented in labor [5]. For simplifying assumptions, no delay in the availability of the GBS culture was assumed. We assumed that the culture is 100% sensitive in identifying colonized women and that antibiotic prophylaxis would be given in a timely fashion with the goal of two doses of penicillin (i.e., duration ≥4 hours). Since penicillin is the agent of choice for intrapartum prophylaxis, and is preferred due to a narrower spectrum of antimicrobial activity, an alternative analysis utilizing ampicillin was not performed [1]. We estimated that 55% of women would receive ≥4 hours of intrapartum antibiotics [6, 7]. We excluded from our calculations the costs to treat maternal postpartum infections since our focus was on infant-related costs [8]. Cost estimates do not include potential costs of prolonging the hospital stay (>48 hours postdelivery) for extended observation or treatment of infants with signs of sepsis.

### 2.2. Intrapartum Prophylaxis in Patients Reporting a Penicillin Allergy

Three percent to eight percent of individuals in the population report an allergy to penicillin [9, 10]. The prevalence of patient-reported penicillin allergy among GBS-positive women is 8% [11]. We assumed that 8% of women in our cohort would report an allergy to penicillin, necessitating prophylaxis with a different antibiotic. Current CDC guidelines recommend that in women with a history of penicillin allergy, assessment should be undertaken to determine whether a high risk for anaphylaxis is present [1]. Those considered at low risk for anaphylaxis may be given cefazolin without further GBS susceptibility testing. Whereas penicillin-allergic patients at high risk for anaphylaxis should have GBS screening with GBS isolates being tested for resistance to clindamycin and erythromycin. If GBS is susceptible to both clindamycin and erythromycin, then either of these antibiotics may be utilized. If the antimicrobial sensitivity is unknown or if GBS is resistant to clindamycin or erythromycin, then vancomycin should be given [1]. 

Since publication of the 2002 CDC guidelines, the largest study looking at intrapartum GBS prophylaxis in patients reporting a penicillin allergy, noted of the women who received appropriate antibiotic prophylaxis, 38% were considered low risk for anaphylaxis and given a cephalosporin [11]. Of the 62% of women considered at a high risk for anaphylaxis, 65% of these women had prophylaxis with vancomycin. The remaining 35% would have been eligible for clindamycin or erythromycin. We therefore made the assumption that 38% of women who reported a history of an allergy to penicillin who either screened positive for GBS or were to be given prophylaxis based on past colonization in a previous pregnancy would be considered low risk for anaphylaxis and treated with a cephalosporin without GBS isolates being tested for susceptibility. Of the 62% of women considered at a high risk of anaphylaxis, GBS screening would be performed (both in the screen and treat group, and the universal treatment group), and those colonized with GBS would have susceptibility testing performed. Antibiotic prophylaxis would be directed as recommended by the CDC guidelines [1]. Although susceptibility testing is recommend for all penicillin-allergic women at high risk of anaphylaxis, only 11% of samples are reported to have this performed [11]. We estimated a midpoint of this range (11%–100%), or a probability of 0.56 for susceptibility testing to be done. Based on the work of Matteson et al. [11], we assumed that 65% of these women would have prophylaxis with vancomycin and the remaining 35% would be eligible for clindamycin or erythromycin.

### 2.3. Effectiveness of Each Strategy

The primary measures of effectiveness of each strategy were (1) the number of early onset neonatal GBS sepsis cases prevented, (2) the total cost of intervention, and (3) overall cost-effectiveness. The baseline efficacy of intrapartum antibiotic prophylaxis, probabilities for each strategy, and outcomes were derived from a systematic review of the English literature, supplemented by bibliographic review. We examined reference lists from studies identified as potentially relevant. The following search terms were used: “group B streptococci,” “group B *streptococcus*,” “*streptococcus* agalactiae,” “pregnancy,” “early onset neonatal group B streptococcal infection,” “maternal colonization with *streptococcus* B,” and combinations thereof, and the databases queried (January 1966 to January 2009) include MEDLINE, PubMed, Scopus, Knowledge Finder, Cochrane database, and the Centers for Disease Control and Prevention guidelines. The probabilities included in the decision tree are shown in Table 1. Reporting guidelines for cost-effectiveness analyses were utilized [24]. We performed sensitivity analyses to account for uncertainty around various measures. Studies utilized for probability estimates were evaluated for their level of evidence as recommended by the United States Preventive Services Task Force [25].

### 2.4. Birth Cohort

A societal perspective for this cost analysis was performed. In 2007, there were 4 315 000 live births in the United States of which 2 597 630 (60.2%) were a second child or greater [26, 27]. Of the total live births, 10.9% are estimated to be repeat cesarean deliveries [28], 12.8% preterm deliveries <37 weeks gestation [27], 0.4% with a previous infant with GBS disease [19], and 2.0% with GBS bacteriuria during the current pregnancy [19, 22]. Thus a potential group of 1 748 719 women greater than 37 weeks gestation with a previous vaginal delivery without a previous infant with GBS disease or GBS bacteriuria in the current pregnancy will present for labor. It has been estimated that greater than 90% of women are screened for group B *streptococcus* in pregnancy [1]. Depending upon the population screened, colonization rates for GBS have been reported to range from 10% to 30% [2]. Assuming that 90% of women were screened in the previous pregnancy and 20% screened positive, then of the 1.7 million multiparous women presenting in labor, 314 769 may have been colonized with GBS in their past pregnancy. For ease of calculations, we rounded this cohort to 300 000 women. Five percent to 11% of women, who are intended to receive prophylaxis for GBS colonization, will not receive it at the time of delivery [11, 19]. We assumed that noncompliance with intrapartum antibiotics would be the same whether the woman presented with known GBS colonization or with intent to treat based on past pregnancy colonization. We therefore estimated a midpoint of this range (5%–11%), or 8% of women would not receive planned intrapartum antibiotic prophylaxis.

### 2.5. Probability Estimates

Table 1 lists the probability estimates used in our analyses and the range cited in the literature. We estimated 0.41 as the probability of recurrence of GBS colonization in a subsequent pregnancy. Cheng et al. [3] demonstrated a rate of recurrence of 0.38 as measured by vaginal-rectal culture in women colonized with GBS in a subsequent pregnancy. Our prior study showed a similar rate of recurrence (0.44) in subsequent pregnancies if GBS colonization was determined by vaginal-rectal culture, and the rate increased to 0.53 if previous colonization was identified from urine and vaginal-rectal culture [4]. Since in the current study we limited our analysis to women without GBS bacteriuria in the subsequent pregnancy, we estimated a midpoint of this range as determined by vaginal-rectal culture (38% to 44%) or a probability of 0.41. 

It has been estimated that a colonized gravida who did not receive intrapartum antibiotics would have a probability of 0.016 delivering a neonate with early onset neonatal GBS [8]. A colonized gravida treated with intrapartum antibiotics has a very low risk of delivering an affected infant. Based on previous published studies, an infant delivered from a GBS colonized mother who received intrapartum antibiotic prophylaxis would have a probability of 0.001 of developing early onset GBS sepsis [8, 12–17]. It has been assumed that only infants of colonized women are at risk for early onset neonatal sepsis, although the birth of colonized infants to culture-negative mothers has been reported [12, 13, 29]. The probability of developing early onset neonatal GBS sepsis if the mother is not colonized and received no intrapartum antibiotics ranges from zero to 0.0004 [15]; however, we utilized 0.0002 as suggested by Benitz et al. [18]. It has been calculated that a 16-fold decrease in early onset neonatal GBS sepsis is observed in infants born to colonized mothers treated with intrapartum antibiotics [8]. We assumed that at a minimum, a similar decrease in the probability of early onset GBS sepsis would theoretically occur in a mother not colonized with GBS who was treated with intrapartum antibiotics yielding a probability of delivering an infant with early onset neonatal GBS of 0.00001. The probabilities estimated for the risk of maternal anaphylaxis to antibiotics were as follows: penicillin 0.000205 [9, 20], cephalosporin 0.00021 [20, 21], and vancomycin 0.0002 [21]. Reports of maternal anaphylaxis to clindamycin or erythromycin are rare. When drug induced anaphylaxis is compared, the ratio of penicillin to erythromycin is 3 to 1 [30]. We therefore assumed that the rate of an anaphylaxis reaction to erythromycin would be 3 times less than the risk from penicillin or a probability of 0.00007. Estimates of anaphylaxis to clindamycin in the general population are lacking [30]. We assumed that the rate would not be greater than that seen with erythromycin, or 0.00007. Maternal deaths from an anaphylactic reaction were assumed to be similar regardless of the drug which initiated the event. We therefore utilized the reported rate for penicillin of 0.0000175 [9].

### 2.6. Cost Estimates

Estimates for direct medical costs (Table 2) were obtained from the literature and were adjusted for inflation to U.S. dollars in 2008 [31]. It was assumed that a vaginal-rectal culture would be performed for screening [8, 18, 32–34]. Women colonized with GBS who reported an allergy to penicillin and were at high risk for anaphylaxis would have susceptibility testing performed [1, 11, 34]. The costs of maternal antibiotic prophylaxis were based on the costs of 5 million units of penicillin G, followed by a second dose of 2.5 million units. For patients reporting an allergy to penicillin, cost estimates were based on a single dose of the following regimens: 2 grams of cefazolin, 1 gram of vancomycin, or 900 mg of clindamycin. For erythromycin we assumed that 95% would deliver within 6 hours and would receive only one dose of 500 mg [35]. Cost sensitivities were performed for women eligible for clindamycin or erythromycin as follows: 100% receiving clindamycin, 100% receiving erythromycin, or half receiving each. Antibiotic costs were the average wholesale cost [36]. Although the average wholesale cost is intended to represent the drug price for transactions between wholesalers and purchasers, it may not take into account all of the discounts and rebates negotiated between the parties in such transactions [37]. Hospital charges for incidental supplies (such as intravenous tubing, diluents, alcohol swabs, and personnel costs) may have a large variability as well. We therefore utilized a sensitivity analysis of 50% more or 50% less of the reported antibiotic costs to account for these unknown variability's. Costs from the medical literature and fee schedules from government sponsored insurances were utilized for estimates of the neonatal complete blood count with differential and blood culture [22, 34, 38–40]. Costs to treat maternal complications were calculated from probabilities and costs of anaphylaxis and maternal death reported in the literature [8, 33, 38, 39]. The total expected, direct, and indirect costs for each case of early onset neonatal GBS sepsis in term infants were obtained from the previous reported estimates [38, 39, 41, 42]. 

Past cost analyses have included expenses associated with cases of neonatal sepsis, but they have not incorporated costs for the pediatric care received by newborns as a result of GBS prevention strategies [8, 18]. It has been suggested that such costs should be included in future cost-effectiveness analyses [22]. Costs for newborns and their mothers treated with antibiotics exceed those of mothers and infants not given antibiotics [38, 39, 43]. The majority of the increasing costs are due to the CDC guidelines proposing that healthy term infants whose mothers received <4 hours of intrapartum antibiotic prophylaxis for GBS should be hospitalized for 48 hours [38, 39]. Pediatric costs of asymptomatic term infants born to mothers receiving intrapartum prophylaxis under a culture-based approach were sensitive to changes in the median length of hospitalization and daily physician assessments [22, 38, 39]. However, since publication of past cost-effectiveness analyses, median length of hospitalization for vaginal deliveries has changed. In 1999, federal legislation required insurers to cover up to 48 hours of hospitalization after a vaginal delivery [44]. Studies comparing the length of stay for infants delivering vaginally whose mother received intrapartum antibiotics for GBS prophylaxis compared with those who did not, demonstrated no significant difference [6, 23]. Since 2000, length of stay for women delivering vaginally and their “well infants” has averaged 2.1 days [45]. We therefore made the assumption that the length of hospitalization in women undergoing a vaginal delivery of a healthy neonate would not be significantly different whether intrapartum antibiotic prophylaxis was given or not. 

### 2.7. CDC Guidelines' Effect on Neonatal Care

In 2002, the CDC proposed revised guidelines for the management of a newborn whose mother received intrapartum antibiotics for prevention of early onset GBS disease [1]. These guidelines recommend that pediatricians observe asymptomatic full-term infants for 24 to 48 hours (dependant upon discharge criteria) if their mother received >4 hours of intrapartum antimicrobial prophylaxis. If mothers at risk receive <4 hours of intrapartum antibiotics, pediatricians should order a complete blood count, a blood culture, and observe infants for 48 hours (i.e., a limited evaluation). A full diagnostic evaluation and empiric therapy is initiated only if sepsis is suspected. Although the algorithm recommends that only neonates with symptoms of sepsis undergo a diagnostic workup, clinicians are more likely to pursue diagnostic testing if the mother received prophylactic intrapartum antibiotics, regardless of the infant's signs and symptoms [23]. Practitioners managing newborns noted that among infants with no signs of sepsis, 20% of neonates whose mothers received intrapartum antibiotics had a CBC, compared with 4% of neonates whose mothers did not receive intrapartum antibiotics [23]. Further, only 4% of infants born to mothers who received intrapartum antibiotics, regardless of whether it was less or greater than 4 hours before delivery, had blood cultures [23]. Since studies suggest that a limited diagnostic workup varies regardless of whether a mother received <4 hours of intrapartum antibiotics, we estimated a midpoint of this range (4% to 100%) or a probability of 0.52 that both a CBC and blood culture would be obtained [1, 6, 23]. If >4 hours of intrapartum antibiotics were received, we assumed that no additional diagnostic evaluation would be performed except for the usual 48 hours of newborn observation.

### 2.8. Cost-Effectiveness Analysis

In the baseline analysis, pathway probabilities were used to calculate costs, number of cases of early onset GBS sepsis prevented, and number of neonatal deaths and morbidity prevented by each strategy. A cost-utility analysis was also performed and reported as cost-effectiveness ratios. The incremental cost-effectiveness ratio represents supplementary costs of changing from a paradigm of using screening with treatment of positive results versus treatment of all previously GBS positive women. The number of life-years was set at 80.4 years or 31.1 years with 3% discounting [46]. Term GBS cases with long-term sequelae attributable to GBS infection are estimated to be 0.016 [38, 39]. In adjusting for long-term sequelae of early onset GBS disease in a term infant, a health-related quality of life score of 0.972 was utilized from prior studies which resulted in an estimate of 78.1 years, and with 3% discounting, this number becomes 30.2 years [42]. 

To calculate the cost per case prevented, we divided the intervention costs by the number of cases of neonatal GBS disease prevented. The benefit-cost ratio was calculated by dividing the monetary savings from preventing disease by the intervention costs. The total costs expected for each strategy were calculated by subtracting the cost for treatment of neonatal GBS disease from the intervention costs of neonatal GBS disease. To determine the expected net benefits, we compared total costs with the costs of treating cases of neonatal GBS disease in the cohort of women if no prevention strategy is used. Data were managed using Excel spreadsheet software (Microsoft Corp, Redmond, WA). 

## 3. Results

In the absence of screening or risk-based strategies, 2003 of the 300 000 newborns were estimated to have early onset GBS disease, of which 60 (3%) would have died [8, 42, 47], and of the remaining survivors, 31 (1.6%) would have long term sequelae [38, 39, 42]. Total cost for treatment of the 2003 term GBS sepsis cases would be $163 442 797 (range $135 731 292 to $191 152 299, are Table 2). Using the baseline assumptions, the model favors universal treatment of term women with intrapartum antibiotic prophylaxis for GBS if they were colonized in a previous pregnancy as the most cost-effective strategy as compared to screening and treating only positive culture results. The costs per quality-adjusted life-year gained from each strategy are shown in Table 3. The conclusions were not affected by varying the antibiotic regimen utilized in women at high risk of anaphylaxis (using either clindamycin or erythromycin) or if all women eligible for susceptibility testing for GBS had this performed (data not shown). Universal treatment with intrapartum antibiotics resulted in an incremental cost *savings* of $209 988 (range $113 920 to $329 258) per quality-adjusted life-year gained when compared to treatment directed by screening. 

The cost for each case of early onset GBS sepsis prevented using intrapartum antibiotic prophylaxis directly was $8805 (range $5 639 to $12 998), compared with $12 710 (range $7773 to $19 105) for women screened then treated. The annual cost savings for the cohort of women in 2007 with direct intrapartum treatment in the U.S. would be $6.35 million (range $3.45 to $9.97 million). When sensitivity analyses were performed for the incidence of recurrent GBS colonization in a subsequent pregnancy, universal treatment remained cost-effective to an incidence as low as 10%. A sensitivity analysis was performed with identical baseline assumptions but presumed that all infants receiving <4 hours of intrapartum antibiotics would have a complete blood count and blood culture, and all penicillin-allergic patients at high risk for anaphylaxis would have GBS screening with GBS isolates being tested for resistance to clindamycin and erythromycin (i.e., strict adherence to the CDC algorithm). Universal treatment with intrapartum antibiotics resulted in an incremental cost *savings* per quality-adjusted life-year gained of $154 063 (range $84 804 to $226 553) when compared to treatment directed by screening. The cost for each additional case of early onset GBS sepsis prevented using intrapartum antibiotic prophylaxis directly was $10 226 (range $6371 to $15 708), compared with $13354 (range $8108 to $20 322) for women screened then treated with an annual cost savings for this cohort of $4.96 million (range $2.73 to $7.29 million).

We considered the results of altering the cost of each variable in Table 2 over the range of costs estimated from the medical literature. Using the baseline assumptions, the model always favored universal treatment of term women with intrapartum antibiotic prophylaxis for GBS if they were colonized in a previous pregnancy as the most cost-effective strategy as compared to screening and treating only positive culture results. Only in the scenario where all infants who received <4 hours of intrapartum antibiotics had a limited diagnostic evaluation (i.e., strict adherence to the CDC algorithm) did the cost of a GBS culture impact the cost-effectiveness of universal (direct) treatment when compared to screening-directed treatment. In this ideal situation, if the cost of a GBS culture became $33 or less, screening-directed treatment would be more cost-effective.

## 4. Discussion

This analysis illustrates that universal treatment in subsequent pregnancies of women with a history of GBS colonization is a more cost-effective strategy for reducing neonatal early onset GBS disease. Administration of intrapartum antibiotics to women with history of colonization in past pregnancies could prevent substantial disease and save millions of dollars annually compared to the current screening-directed treatment approach. 

Cost-effectiveness analysis has been advocated as a basic tool in the evaluation of health care practices [48]. When uncertainty exists about the appropriate clinical strategy for patients with a given health state, or when a randomized prospective trial is not feasible, policy makers may utilize cost-effectiveness analysis to guide decisions for intervention. Current treatment guidelines from the CDC and ACOG recommend that women colonized with GBS in previous pregnancies undergo universal antenatal culture-based screening at 35 to 37 weeks of gestation to direct treatment for the prevention of early onset GBS disease [1, 2]. However, despite these recommendations, some obstetricians administer antibiotic prophylaxis based on prior history of GBS colonization [4, 5]. When surveyed, obstetricians in the U.S. have shown a preference for strategies for GBS prophylaxis not described in consensus guidelines with the most common strategy providing intrapartum antibiotics to all GBS colonized women and women with negative screening cultures who develop risk factors [49]. The optimal decision in this scenario should be based on a randomized controlled trial. However, to detect a difference in the rate of early onset GBS sepsis between the two strategies compared would require over 5000 women in each arm of the trial.

Specific concerns about universal antibiotic prophylaxis hinge on the possibility of adverse antibiotic reactions and the development of antimicrobial resistance [33]. Nevertheless, the strategy of universal treatment is based on past GBS colonization in which most women would have received antibiotics in the previous pregnancy. The incidence of anaphylaxis from an initial penicillin exposure compared to a subsequent exposure have not been shown to be different (0.1 per 10 000 dispensing) [20]. However, case reports of women given intrapartum prophylaxis for GBS who received previous penicillin-related antibiotics without allergic reactions have had subsequent episodes of anaphylaxis [50]. In addition, the costs of treating other antibiotic associated adverse medical events such as pseudomembranous colitis were not included in the current analysis. Past studies have not shown an increase in these events when antibiotic prophylaxis was introduced for neonatal GBS disease prevention [6]. Theoretical risk of selecting antibiotic-resistant GBS or alteration of neonatal flora allowing for early onset neonatal sepsis attributable to other pathogens such as *Escherichia coli* has been raised [8]. With more than 40% of mothers receiving intrapartum antibiotics for GBS prevention, cesarean prophylaxis, or clinical chorioamnionitis [43], emerging resistance of GBS to erythromycin and clindamycin has been reported [51]. Despite the appearance of increasing number of resistant GBS strains, GBS remains universally sensitive to penicillin [51]. Exposure to ß-lactam antibiotics (either ampicillin or penicillin) for intrapartum GBS prophylaxis has been associated with increases in postpartum ampicillin-resistant organisms [52]. However, despite the common use of intrapartum antibiotic prophylaxis, increased rates of early onset neonatal infection with ampicillin-resistant gram-negative organisms has not been seen in term infants, and moreover, intrapartum antibiotic prophylaxis has shown a protective effect [53]. This current data suggest that the potential tradeoff of widening the scope of intrapartum antibiotic administration would not impact the rate of early onset neonatal sepsis in term infants attributable to other pathogens.

Our study has several limitations. The current analysis focuses on the prevention of early onset GBS neonatal sepsis. No costs or outcomes were included to treat maternal pregnancy-associated disease or for infants who developed late-onset GBS disease. However, the incidence of late-onset GBS neonatal sepsis has not been shown to be impacted by intrapartum antibiotic prophylaxis [47].The present analysis is limited by estimates of cost from the medical literature and fee schedules from government sponsored insurances. The utilization of the average wholesale price for estimates of drug costs has been the subject of extensive criticism for its failure to reflect actual prices paid in the market [37]. Values reported in the medical literature for two doses of penicillin which included costs for supplies and personnel (adjusted for inflation to 2008), range between $38 to $48, similar to the range of our estimate [32, 38, 39]. However, sensitivity analyses were performed over a wide array of cost values available. We assumed that obstetricians would have access to the results of a patient's GBS status from her prior pregnancy. With the integration of electronic medical records in obstetrics and patient education of the impact of past pregnancy GBS colonization, knowledge of this in future pregnancies could be available. In addition, we assumed that in women undergoing an uncomplicated term vaginal delivery that the length of hospitalization would not differ based on receiving intrapartum antibiotics. Studies comparing average length of stay for newborns whose mothers were treated with intrapartum antibiotics have had conflicting results [6, 23, 43]. However these studies predominately evaluated hospital length of stay prior to the implementation in 1999 of federal legislation requiring insurers to cover up to 48 hours of hospitalization after a vaginal delivery [44]. Since 2000, the length of stay for women with vaginal deliveries and their “well infants” has averaged >48 hours [40]. This average length of stay did not change after implementation of CDC guidelines for GBS prophylaxis in 2002. Finally, the results of cost-effectiveness analysis rest on the baseline assumptions put into the model which is limited by the quality and quantity of the available information. Since no measures of inherent variability (i.e., probabilities with confidence intervals) in the form of statistical analyses are utilized, one must interpret the results of cost-effectiveness analysis with this in mind. 

## 5. Conclusions

Our analysis suggests that in this well defined population, universal intrapartum antibiotic prophylaxis in future pregnancies of women with GBS colonization in a prior pregnancy could prevent disease and represent a cost-savings compared to current screening strategies for prevention of early onset GBS infection.

## Figures and Tables

**Table 1:** Probability estimates used in the decision analysis.

Variable	Baseline estimate	Range for sensitivity analysis	References	Level of evidence*
Probability of being colonized with GBS in a subsequent pregnancy				
	0.41	0.38–0.44	[3, 4]	II
				
Probability of early onset neonatal GBS sepsis if mother colonized and treated				
	0.001	0–0.011	[12–17]	I
				
Probability of early onset neonatal GBS sepsis if mother colonized and not treated				
	0.016	0.011–0.066	[8, 13–17]	I and II
				
Probability of early onset neonatal GBS sepsis if mother not colonized and given no treatment				
	0.0002	0–0.0004	[8, 18]	II
				
Probability of early onset neonatal GBS sepsis if mother not colonized and given treatment				
	0.00001	0–0.0004	See methods	
			
Probability of not receiving planned antibiotic prophylaxis				
	0.08	0.05–.11	[11, 19]	II
				
Probability of maternal anaphylaxis to penicillin				
Penicillin	0.000205	0.00001–0.0004	[9, 20]	II and III
Cephalosporin	0.000215	.000015–0.0004	[20, 21]	II and III
Clindamycin	0.00007	0.000003–0.00013	See methods	
Erythromycin	0.00007	0.000003–0.00013	See methods	
Vancomycin	0.0002	0.00001–0.0004	[21]	III
				
Probability of maternal death from antibiotic anaphylaxis				
	0.0000175	0.000015–0.00002	[9]	III
				
Probability of individual reporting an allergy to penicillin at high risk of anaphylaxis colonized				
with GBS and having susceptibility testing done				
	0.56	0.11–1	[11]	II
				
Culture positive gravidas receiving ≥4 hours intrapartum antibiotics				
	0.55	0.53–0.65	[6, 7, 22]	II and III
				
Probability of a newborn limited diagnostic workup born to mothers colonized with GBS				
who received <4 hours antibiotics before delivery				
	0.52	0.04–1	[1, 6, 23]	II and III
				
Probability of obtaining a newborn CBC in neonates born to mothers negative for				
GBS colonization				
	0.07	0–0.14	[6, 23]	II

GBS: group B streptococci; CBC: complete blood count.

*US Preventive Services Task Force (10). Level I is the best evidence, and III is the worst evidence.

**Table 2:** Costs (in 2008 USD) included in decision tree.

Variable	Baseline estimate	Range for sensitivity analysis	Source or references
Cost of GBS rectal-vaginal culture			
	$50	$30–$75	[8, 18, 32, 34, 41]
			
Cost of GBS rectal-vaginal culture with susceptibility testing			[34]
	$59	$35–$89	[36]
			
Cost of maternal antibiotic therapy			
One dose of 5 million units of penicillin G			
	$18	$9–$27	
Second dose of 2.5 million units of penicillin G			
	$11	$6–$17	
			
One dose of 2 grams of cefazolin			
	$14	$7–$21	
One dose of 900 milligrams of clindamycin			
	$18	$9–$27	
One dose of 500 milligrams of erythromycin			
	$16	$8–$24	
One dose of 1 gram of vancomycin			
	$12	$6–$18	
			
Cost of neonatal CBC			[22, 34, 38, 39]
	$15	$8–$41	
			
Cost of neonatal blood culture			[22, 34, 40]
	$20	$10–$26	
			
Cost of maternal anaphylaxis case			
	$11 160	$1186–$30 286	[8, 33, 38, 39]
			
Cost of maternal death due to anaphylaxis			
	$1 211 432	$1 090 288–$1 332 575	[8]
			
Direct cost of early onset GBS sepsis case term infant			
	$19 774	$17 723–$21 824	[38, 39, 41]
			
Total (direct and indirect) cost of early onset GBS sepsis case term infant			
	$81 599	$67 764–$95 433	[38, 39, 42]

GBS: group B streptococci; CBC: complete blood count.

**Table 3:** Estimated effects and costs (in 2008 USD) of the different treatment strategies compared with a situation without treatment for a birth cohort of 300 000 infants born to mothers colonized with GBS in a previous pregnancy (3% discounting).

	Screen and treat strategy		Universal treatment strategy	
*Effects*				

EOGBS cases prevented	1700		1732	
Deaths due to EOGBS prevented	51		52	
QALY gained	1607		1637	

		Range*		Range*
*Cost (in dollars)*				

Costs of screening	15 000 000	9 000 000–22 500 000	NA	
Cost of IAP with penicillin	2 503 778	1 280 519–3 784 297	6 106 776	3 123 216–9 229 992
Cost of screening and treating women with a history of a penicillin allergy	154 551	79 011–233 562	223 856	113 664–337 519
Treatment for maternal anaphylaxis and death	2 658 264	2 188 173–3 339 396	6 293 596	5 178 717–7 910 099
Cost of limited evaluation of well infants whose mothers received <4 hours of IAP	1 104 569	568 064–2 114 460	2 617 008	1 345 890–5 009 702
Cost of CBC of well infants whose mother was negative for GBS	185 850	99 120–507 990	9 367	4 996–25 603
Total costs for treatment of term GBS sepsis cases	−24 719 871	20 528 650–28 910 788	−22 111 508	18 362 532–25 860 213
Total costs of intervention	21 607 013	13 214 887–32 479 706	15 250 603	9 766 483–22 512 915
Numbers needed to treat per GBS case prevented	67		155	

*Cost-effectiveness (in dollars)*				

Cost savings per QALY gained	72 878	78 100–66 112	77 006	80 355–72 570
Cost for each GBS case prevented	12 710	7 773–19 105	8 805	5 639–12 998
Benefit-cost ratio	6.4	10 5–4.3	9.3	14.5–6.3
Total annual costs net benefits	141 835 784	150 227 910–130 963 091	148 192 194	153 676 314–140 929 882

EOGBS: early onset group B streptococci; QALY: quality-adjusted life-year gained; IAP: intrapartum antibiotic prophylaxis; GBS: group B streptococci; CBC: complete blood count. NA: not applicable.

*Range: it is the cost utilizing the baseline probability estimates from Table 1 and the lowest to the highest estimates shown in Table 2.
